# Electronic Health Record Patient Portal Adoption by Health Care Consumers: An Acceptance Model and Survey

**DOI:** 10.2196/jmir.5069

**Published:** 2016-03-02

**Authors:** Jorge Tavares, Tiago Oliveira

**Affiliations:** ^1^ NOVA Information Management School (IMS) Universidade Nova de Lisboa Lisboa Portugal

**Keywords:** UTAUT2, technology adoption, eHealth, health care consumers, electronic health records, technology acceptance

## Abstract

**Background:**

The future of health care delivery is becoming more citizen centered, as today’s user is more active, better informed, and more demanding. Worldwide governments are promoting online health services, such as electronic health record (EHR) patient portals and, as a result, the deployment and use of these services. Overall, this makes the adoption of patient-accessible EHR portals an important field to study and understand.

**Objective:**

The aim of this study is to understand the factors that drive individuals to adopt EHR portals.

**Methods:**

We applied a new adoption model using, as a starting point, Ventkatesh's Unified Theory of Acceptance and Use of Technology in a consumer context (UTAUT2) by integrating a new construct specific to health care, a new moderator, and new relationships. To test the research model, we used the partial least squares (PLS) causal modelling approach. An online questionnaire was administrated. We collected 360 valid responses.

**Results:**

The statistically significant drivers of behavioral intention are performance expectancy (beta=.200; *t*=3.619), effort expectancy (beta=.185; *t*=2.907), habit (beta=.388; *t*=7.320), and self-perception (beta=.098; *t*=2.285). The predictors of use behavior are habit (beta=0.206; *t*=2.752) and behavioral intention (beta=0.258; *t*=4.036). The model explained 49.7% of the variance in behavioral intention and 26.8% of the variance in use behavior.

**Conclusions:**

Our research helps to understand the desired technology characteristics of EHR portals. By testing an information technology acceptance model, we are able to determine what is more valued by patients when it comes to deciding whether to adopt EHR portals or not. The inclusion of specific constructs and relationships related to the health care consumer area also had a significant impact on understanding the adoption of EHR portals.

## Introduction

### Overview

Our study focuses on a specific type of eHealth technology, the patient-accessible electronic health record (EHR) portals [1-5]. To better understand the definition of EHR portals it is important to have a clear view of the technologies that support them. First are the patient portals, health care-related online applications that allow patients to interact and communicate with their health care providers [3,5]. The second is the EHR, meaning a repository of patient data in digital form, stored and exchanged securely. EHR systems are the software platforms that physician offices and hospitals use to create, store, update, and maintain EHRs for patients [2]. By definition, an EHR portal is a Web-based application that combines an EHR system and a patient portal, not only for patients to interact with their health care providers, but also to access their own medical records and medical exam results [2-7].

EHR portals may help patients carry out self-management activities, thereby making the use of the health care system more effective and sustainable, not only from the patient care standpoint, but also from a financial perspective due to rising health care costs and budgets in many countries [8-11]. A recent survey of US health care providers shows that 57% of health care institutions already have a portal in place and 71% value the integration of the EHR system within the patient portal by choosing a product (ie, patient portal interface) from their EHR vendor [7]. In Europe, not only health care providers, such as hospitals and clinics, provide EHR portals, but also governmental institutions make these platforms available to patients [8,12].

This concept of a national-level patient portal progressed into a trans-European initiative, the European Patients Smart Open Services (epSOS). epSOS concentrates on developing a practical eHealth framework, and an information and communication technology (ICT) infrastructure that enables secure access to patient health information among different European health care systems [13]. The pilot stage of this project, which ended in June 2014, focused on cross-border eHealth services in the following areas: patient summary and cross-border use of electronic prescriptions [13]. In the United States, a new guidance was issued by the Centers for Medicare & Medicaid Services (CMS) called Stage 2 meaningful use [5,14]. This guidance requires that the eligible professionals and hospitals that participate in the Medicare & Medicaid EHR Incentive Programs must give their patients secure online access to their health information, including EHRs [5,7,14]. Stage 2 meaningful use boosted the development of new integrated EHR portals in the United States by health care providers that, according to the new guidance, must not only implement it but also demonstrate effective use by the patients [5,7,14]. According to the literature, the most used features in EHR patient portals are as follows: scheduling medical appointments, email messaging, requesting prescription refills, and checking of patients’ medical exams [1,3,15].

The aim of this study is to identify a set of determinants in the adoption of electronic health record portals by health care consumers. In our study, we examine these determinants in the field of eHealth technology use and acceptance by health care consumers. We then propose a new research model based on Venkatesh's Unified Theory of Acceptance and Use of Technology in a consumer context (UTAUT2) by integrating a new construct from the health care area, self-perception (SP), and a new moderator, chronic disability (CD) [2,16-18].

In this paper, we first review the literature concerning information technology (IT) adoption models regarding consumer health care. We then present a research model to analyze EHR portals for the health care consumer. Finally, we discuss the issue and present conclusions.

### Theoretical Background

There have been several theoretical models developed from theories in psychology, sociology, and consumer behavior employed to explain technology acceptance and use [18]. The goal of this study is to focus specifically on EHR portal adoption from the perspective of the health care consumer, so it is of the utmost importance to review the literature in this particular field. Adoption of eHealth technologies by patients is clearly a very important topic in information systems (IS) in health care. The adoption of eHealth technologies by health care consumers still requires more attention and research due to the limited number of studies reported in the literature to date [2,19-22]. The use of the UTAUT2 model might be beneficial to eHealth adoption due to its consumer-specific constructs like price value [21].

**Figure 1:**
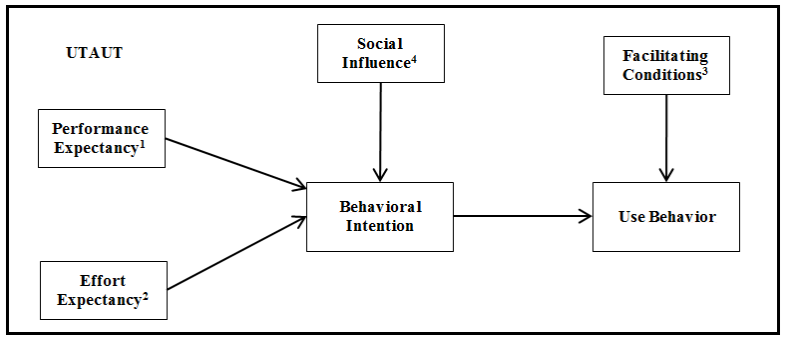
Unified Theory of Acceptance and use of Technology (UTAUT) model adapted from Venkatesh et al [35]. Notes: 1. Moderated by age and gender; 2. Moderated by age, gender, and experience; 3. Moderated by age and experience; 4. Moderated by age, gender, experience, and voluntariness of use.

When studying eHealth and health care adoption by health care professionals, the most common adoption models used are the technology acceptance model (TAM) [23,24] and the unified theory of acceptance and use of technology (UTAUT) [25-29]. Evaluating the studies published in the field of consumer health IT adoption, and more specifically in the use and adoption of eHealth tools by the health care consumer, most studies use TAM or extensions of TAM [19,30-34]. TAM was designed and tailored in IS contexts to predict information technology acceptance and usage on the job. TAM uses three dimensions: perceived usefulness (PU), that is “the degree to which a person believes that using a particular system would enhance his or her job”; perceived ease of use (PEOU), that is “the degree to which a person believes that using a particular system would be free of effort”; and attitude toward technology use [32,35,36]. PU and PEOU together affect the attitude toward technology use, which in turn influences behavioral intention to adopt [32,36]. UTAUT formulates a unified model that integrates elements of eight models in the field of IT acceptance, including from TAM, which incorporates the concept of PU as performance expectancy and PEOU as effort expectancy [35]. Apart from these two constructs from TAM, UTAUT also uses two other constructs, social influence and facilitating conditions (FC). All of these are joined together in the model along with four moderators—age, gender, experience, and voluntariness of use. The model and its relationships are illustrated in Figure 1 [35]. The *R* ^2^ obtained with UTAUT was superior to those of any of the individual models, including TAM, making a synthesis of the different theories by bringing together into the model the constructs that have a significant impact [18,35]. For example, with UTAUT it is possible to measure the impact of social influence on behavioral intention, something that was not measured with TAM [18,35]. Although UTAUT provides better results than TAM and other IS adoption models, the focus of UTAUT is also the employee technology acceptance at the individual level, which is not the focus of our paper because our target group is health care consumers [18].

**Figure 2:**
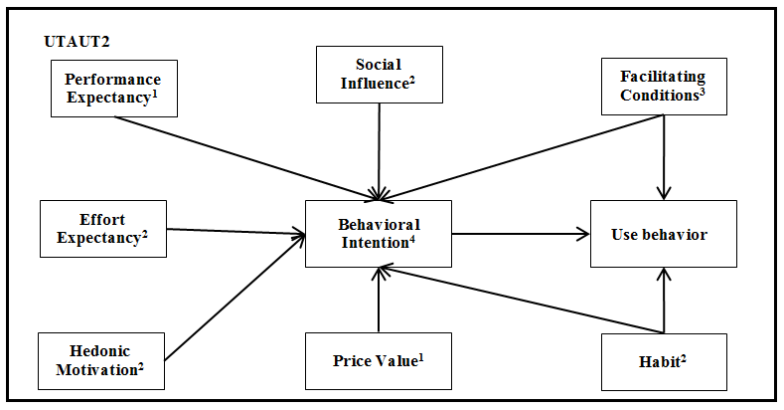
Unified Theory of Acceptance and use of Technology in a consumer context (UTAUT2) model adapted from Venkatesh et al [18]. Notes: 1. Moderated by age and gender; 2. Moderated by age, gender, and experience; 3. Effect on behavioral intention is moderated by age, gender, and experience. Effect on use behavior is moderated by age and experience; 4. Moderated by experience.

Ideally, we need a model tailored to the consumer use context, and in this specific field, UTAUT2 was developed with this goal, obtaining very good results [18,21]. This new model includes the same four UTAUT constructs, but which are moderated differently. The constructs are now moderated only by age, gender, and experience [18]. The moderator *voluntariness of use* was dropped since the target population was not obliged to use the technology [18]. UTAUT2 also introduces three new constructs (ie, specific consumer adoption constructs): hedonic motivation, price value, and habit. Hedonic motivation and price value explain behavioral intention, while habit explains behavioral intention and use behavior [18]. Compared to UTAUT, the extensions proposed in UTAUT2 that are consumer specific produced a substantial improvement in the variance explained in behavioral intention (from 56% to 74%) and technology use (from 40% to 52%) [18]. Including these three new constructs made UTAUT2 a more suitable model for consumer-centered technologies [18]. Figure 2 explains the UTAUT2 model. The definitions of the different constructs used in the UTAUT and UTAUT2 models are provided in the Research Model section of this paper. Most of the existing UTAUT2 literature focuses on other types of technologies, such as online purchasing, mobile banking, and Web-based services [18,37-39]. A recently published study used UTAUT2 in health and fitness apps, which is not exactly the same technology scope and type of eHealth service as EHR portals, but obtained the following results: performance expectancy, hedonic motivation, price value, and habit were significant predictors of intention of continued usage [40].

Table 1 summarizes some of the studies performed in the area of eHealth, the theory or theories behind the studies, the dependent variable that is being explained by each study, and the most important findings. The target population in all studies was patients and the technologies have similarities with EHR portals [2,16,30,31,41,42].

**Table 1:** eHealth adoption models.

Theory	Dependent variable	Findings	Reference
TAM^a^, motivational model (MM), integrated model (IM)	eHealth behavioral intention	Users’ perceived ease of use (PEOU), users’ perceived technology usefulness (PU), intrinsic motivation (IM), and extrinsic motivation (MM) have a significant positive influence on behavioral Intention. IM does not have a better performance than TAM or MM when predicting behavioral Intention.	[30]
Elaboration likelihood model (ELM), concern for information privacy (CFIP)	EHR^b^ behavioral intention	Positively framed arguments and issue involvement generate more favorable attitudes toward EHR behavioral intention. CFIP is negatively associated with likelihood of adoption.	[2]
TAM (qualitative study)	eHealth services behavioral Intention	PU seemed to be important. PEOU did not seem to be an issue. Although experience is not a TAM construct, it seemed to have influenced behavioral Intention.	[41]
TAM, plus several other constructs	Internet use behavior as a source of information	PU, importance given to written media in searches for health information, concern for personal health, importance given to the opinions of physicians and other health professionals, and the trust placed in the information available are the best predictors to use behavior.	[42]
Personal empowerment	Internet use behavior as a source of information	There are three types of attitudes encouraging Internet use to seek health information: professional, consumer, and community logic.	[16]
Extended TAM in health information technology (HIT)	HIT behavioral intention	PU, PEOU, and perceived threat significantly impacted health consumers’ behavioral intention.	[31]

^a^TAM: technology acceptance model.

^b^EHR: electronic health record.

### Research Model

UTAUT2 was developed as an adoption model providing the general factors of IT adoption in consumer use. However, according to Venkatesh et al [18], in certain situations in which the technology may be influenced by specific factors it may be necessary to extend the model with new constructs, moderators, and relationships. We therefore identified key additional constructs and relationships based on the literature review that are specific to IT health care adoption to be integrated into UTAUT2, thus tailoring it to the eHealth consumer context, with the special aim of studying the adoption of EHR portals. We did this by (1) identifying a key construct from earlier research in health care—self-perception—and by (2) adding a new moderator specific to health care use—chronic disability.

**Figure 3:**
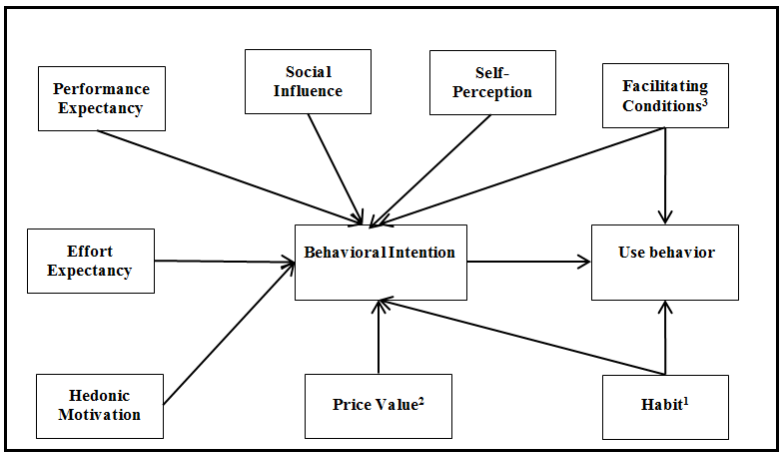
The research model. Notes: 1. Moderated by age or gender; 2. Moderated by age; 3. Moderated by chronic disability on use.

Published studies suggest that patients with chronic illness, severe illness, or disability are more likely to use eHealth technologies if they have the resources and support available [17,43,44]. A national survey in the United States shows that 86% of people living with disability or chronic illness with Internet access have looked online for information about health topics, compared with 79% of Internet users with no chronic conditions [44]. A recent study using a TAM extended version with the health belief model (HBM) measured the perceived health risk to chronic diseases [32]. Using chronic disability with UTAUT2 in the field of EHR portals is not only a new approach, but also one that takes advantage of the existence of the construct facilitating conditions—defined as the individual perception of the support available for using a technology activity [35]—that can be moderated by chronic disability, something that can be more properly tested with UTAUT2 than with TAM [18]. Recent studies tackled the need to study the variables that can drive the patients to be more active in their own health management [8,21]. Self-perception in health [45-47], called the self-perception construct, considers that the perceived, rather than the real, severity of the health complaint could be the propelling force behind the action in health care [45,47,48]. EHR portals are interfaces that links patients with health care professionals, and this construct is relevant to understanding if the patient’s awareness about her/his own health status can be a driver to adopt EHR portals. Other studies using the health belief model with TAM [31,32] incorporated other constructs related to the health belief model concept. One such study was by Kim and Park [31], who studied health-related constructs like health belief and concerns or perceived health status, conceptually similar to self-perception, that have been shown to have an indirect effect on the behavioral intention to use health information technology [31]. This shows the importance of measuring this dimension in our study with a consumer-centered adoption model. Figure 3 illustrates the new research model.

### Research Model: Extended Unified Theory of Acceptance and Use of Technology in a Consumer Context Model

Performance expectancy is defined as the degree to which using a technology will provide benefits to consumers in carrying out certain activities [35,49]. Our literature review indicates that health care consumers tend more to adopt eHealth technologies that provide clear benefits, such as obtaining an electronic medical prescription via EHR portals [8,50,51]. *Hypothesis 1* (H1) states that performance expectancy will positively influence behavioral intention.

Effort expectancy is the degree of ease related to consumers’ use of technology [35]. The easier it is for consumers to understand and use an eHealth technology, the greater is the probability that they will adopt it [8,51]. *Hypothesis 2* (H2) states that effort expectancy will positively influence behavioral intention.

Social influence is the extent to which consumers perceive that others who are important to them (eg, friends and family) believe they should use a particular technology [18]. In the case of eHealth, this can also be an important construct since people who share the same diseases (eg, multiple sclerosis) or the same health condition (eg, obesity) tend to be influenced by others having the same condition [20,52]. *Hypothesis 3* (H3) states that social influence will positively influence behavioral intention.

The construct, facilitating conditions, is defined as the individual perception of the support available for using a technology activity [35]. One of the barriers to consumers’ use of health services over the Internet is the consumers’ lack of resources to access these platforms [51], suggesting that users with better conditions to use eHealth technologies favor EHR portals adoption. *Hypothesis 4 (a)* (H4 [a]) states that facilitating conditions will positively influence behavioral intention.

Chronic disability is an incapacitating situation (eg, chronic illness) that affects a patient permanently or for long-term periods. Our literature review reveals that patients with chronic illness or disability are more likely to use eHealth technologies if they have the resources and support available (ie, facilitating conditions) [17,20]. *Hypothesis 4 (b)* (H4 [b]) states that chronic disability will moderate the effect of facilitating conditions on use behavior, such that the effect will be stronger for chronically disabled people.

Hedonic motivation is defined as intrinsic motivation (eg, enjoyment) and has been included as a key predictor in much of the reported consumer behavior research [18]. Obtaining and dealing with information about our health status by using eHealth technologies may be an enjoyable process, or in some cases may not be when a patient has, for example, an incurable disease [53]. Nevertheless, in a recent study with UTAUT2 in eHealth, hedonic motivation was found to have a significant impact on behavioral intention [40]. We then propose that this specific construct may have a significant impact in predicting EHR portal use. *Hypothesis 5* (H5) states that hedonic motivation will have a positive influence on behavioral intention.

Price value in a consumer use environment is also a relevant factor as, unlike workplace technologies, consumers must bear the costs related with the purchase of devices and services [18]. If a patient can obtain her/his medical prescription via an EHR portal, she/he can save transportation costs by avoiding a trip to a health center or hospital. The better the perception a health care consumer has about the *price value* of an eHealth technology (ie, that it can help save money), the more likely it is that she/he will adopt it [8,11]; older people tend to give more importance to price in eHealth [21]. *Hypothesis 6* (H6) states that age will moderate the effect of price value on behavioral intention, such that the effect will be stronger for older people.

Habit can be defined as the extent to which people tend to execute behaviors automatically because of learning [18]. We can expect that habit will positively influence eHealth adoption, as it does in other IT adoption fields, since habit is a concept that should not be specific to an IT technology [18]. The literature review indicates that in eHealth, younger people and women tend to have the habit to use more eHealth technologies [17,20]. *Hypothesis 7 (a1)* (H7 [a1]) states that age will moderate the effect of habit on behavioral intention, such that the effect will be stronger for younger people. *Hypothesis 7 (a2)* (H7 [a2]) states that gender will moderate the effect of habit on behavioral intention, such that the effect will be stronger for women. *Hypothesis 7 (b1)* (H7 [b1]) states that age will moderate the effect of habit on use behavior, such that the effect will be stronger for younger people. *Hypothesis 7 (b2)* (H7 [b2]) states that gender will moderate the effect of habit on use behavior, such that the effect will be stronger for women.

Behind the concept, self-perception, is the health belief model. The model assumes that subjective health considerations determine whether people perform a health-related action, such as consulting their physician [45]. For example, the health belief model considers the perceived, rather than the real, severity of the complaint to be the propelling force behind the action [45].

Studies about patients that look for information online seem to confirm the concept of the health belief model; the results show that a larger proportion of respondents who described their health as poor indicated that they looked for health-related information online “often” compared with those who described their health as fair or better [54]. We therefore add self-perception as a predictor of health consumer behavioral intention to use a technology. *Hypothesis 8* (H8) states that self-perception will positively influence behavioral intention.

The role of intention as a predictor of usage is critical and has been well established not only in IS in general, but also in health care and eHealth, with the literature suggesting that the driver of using specific eHealth platforms is preceded by the intention to use them [18,22,30,31,35,45]. *Hypothesis 9* (H9) states that behavioral Intention will positively influence use behavior.

## Methods

### Measurement

All of the items were adopted from Venkatesh et al [18], Wilson and Lankton [30], and Vandekar et al [45] with small modifications in order to adjust to EHR portal technology. The items are shown in Multimedia Appendix 1. The questionnaire was administered in Portuguese through a Web hosting service after being translated by a professional translator. In order to ensure that the content did not lose its original meaning, a back-translation was made from the Portuguese instrument to English, again by a professional translator, and compared to the original [55].

The scales’ items were measured on a 7-point Likert scale, ranging from *strongly disagree* (1) to *strongly agree* (7). Use was measured on a different scale. The scale from UTAUT2—from *never* to *many times per day*—was adapted to *never* to *every time I need*, since EHR portal usage is not as regular as mobile Internet usage. Demographic questions about age and gender were also included; age was measured in years and gender was coded as a dummy variable (0 or 1), with women represented by 0. Chronic disability was coded as a dummy variable (0 or 1), with its absence represented by 0.

Before the respondents could see any of the questions, an introduction was made explaining the concept of EHR portals (see Multimedia Appendix 1). The aim of this introduction was to ensure that respondents were aware of this concept and had prior knowledge and contact with EHR portals, because the absence of this prior knowledge is an exclusion criterion.

### Data Collection

A pilot survey was conducted to validate the questions and the scale of the survey. From the pilot survey, we had 30 responses demonstrating that all of the items were reliable and valid. The data from the pilot survey were not included in the main survey.

According to the literature, the technology that we are studying (EHR portals) is being used by less than 7% of the total number of health care consumers or patients [5,7,56]. We are therefore sampling a group of people that could be defined as a rare population, as it constitutes a small proportion of the total population, and specific sample strategies can be used that are suitable in this case [57,58]. We have a disproportionate stratification of our target population compared with the general population, because according to the literature, users and early adopters of these types of platforms have significantly higher education [19,43,59]. As a result, we focused our sampling strategy in places where our target population—users of EHR portals—are more concentrated [57,58]; thus, we selected educational institutions.

The survey, via hyperlink, was sent by email in October 2013 to a total of 1618 people at three institutions that provide educational services, from which we obtained 350 responses. NOVA Information Management School (IMS) approved and verified the ethical compliance of the questionnaire before its use. All participants were informed by email about the study purpose, confidentiality protection, and the anonymity of the information collected. A reminder was sent 2 weeks after the first email, only to those who had not responded to the first email, in order to improve the response rate. Following the reminder, we had a total of 465 respondents out of 1618 (28.74% response rate). After removing the invalid responses, the final sample consisted of 360 respondents. A questionnaire was considered invalid if not all questions were answered. According to our statistical modelling method, we cannot use incomplete questionnaires [60,61].

### Data Analysis

To test the research model, we used the partial least squares (PLS) method, which is a causal modelling approach that represents a variance-based technique of path modelling [60]. Our main reasons for choosing this method were the complexity of the model (ie, many moderators) and the fact that the PLS method is oriented to explain variance of the research model and to identify key constructs [60-62]. We used the software program SmartPLS version 2.0.M3 (SmartPLS GmbH) [63] to estimate the PLS. Before testing the structural model, we examined the measurement model to assess construct reliability, indicator reliability, convergent validity, and discriminant validity.

## Results

### Sample Characteristics

Our sample characteristics are shown in Table 2.

The literature mentions that users of EHR portals are younger than the population average and have significantly higher education [19,43,59]; the results shown in Table 2 are aligned with the literature findings.

### Usage Results

**Figure 4:**
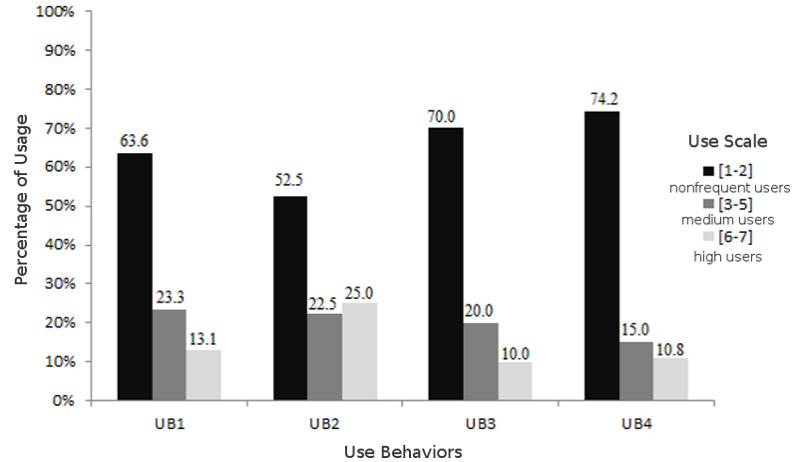
Types of usage patterns of electronic health record (EHR) portals. UB: use behavior; UB1: management of personal information and communication with health providers; UB2: medical appointment schedule; UB3: check their own EHR; UB4: request for medical prescription renewals.

Use was measured on a scale that ranges from *never* (1) to *every time I need* (7). In Figure 4, we grouped the results by nonfrequent users of a particular EHR portal feature (scale from 1 to 2), medium users (scale from 3 to 5), and high users (scale from 6 to 7). These results show that the fact that people know about the technology and enter and register in these portals does not make them frequent users. Our study results are aligned with those of earlier studies and reports [3,12,17]; also, the results from our study show that only 30% of users use a portal regularly to check their EHR. Medical appointment scheduling is the feature with the highest usage.

**Table 2:** Sample characteristics (n=360).

Variable and category		Frequency, n (%)
**Age in years**		
	18-20	69 (19.2)
	21-24	75 (20.8)
	25-30	76 (21.1)
	31-40	89 (24.7)
	>40	51 (14.2)
**Gender**		
	Male	142 (39.4)
	Female	218 (60.6)
**Chronic illness/disability**		
	No	308 (85.6)
	Yes	52 (14.4)
**Education**		
	Undergraduate	132 (36.7)
	Bachelor’s degree	87 (24.2)
	Postgraduate	70 (19.4)
	Master’s degree or more	71 (19.7)

### Measurement Model

The results of the measurement model are shown in Tables 3, 4, and 5 and in Multimedia Appendix 2. To evaluate construct reliability, one can use Cronbach alpha or the composite reliability coefficient (CR). Although Cronbach alpha is more often used, CR is more appropriate for PLS since it prioritizes indicators according to their individual reliability and takes into account that indicators have different loadings, unlike Cronbach alpha [64]. Table 3 reports that all constructs have a CR greater than .70, showing evidence of internal consistency [60,65].

**Table 3:** Cronbach alpha, composite reliability, and average variance extracted.

Construct	Cronbach alpha	Composite reliability coefficient (CR)	Average variance extracted (AVE)
Performance expectancy	.90	.94	.83
Effort expectancy	.91	.94	.79
Social influence	.98	.98	.96
Facilitating conditions	.80	.87	.63
Hedonic motivation	.93	.96	.88
Price value	.93	.96	.88
Habit	.74	.85	.66
Self-perception	.67	.81	.52
Behavior intention	.90	.94	.83

In order to have good indicator reliability, it is desired that the latent variable explain more than half of the indicators’ variances. The correlation between the constructs and their indicators should ideally be greater than .70 (√.50 ≈.70) [60,65]. However, an item is recommended to be eliminated only if its outer standardized loadings are lower than .40 [66]. The measurement model has issues with two indicators’ reliabilities—SP3 and SP5—which were removed; FC4, SP4, and SP6 are lower than .70, but still greater than .40 (see Multimedia Appendix 2).

In order to assess the convergent validity, we used average variance extracted (AVE). The AVE should be greater than .50, so that the latent variable explains, on average, more than 50% of its own indicators [64,67]. As shown in Table 3, all of the indicators respect this criterion. Finally, discriminant validity can be evaluated with the Fornell-Larcker criterion [67]. This criterion claims that a latent variable shares more variance with its indicators than with the other latent variables, so that the square root of AVEs should be greater than the correlations between the construct [60,67]. As seen in Table 4, all diagonal—square root of AVEs—are greater than the correlation between constructs—off-diagonal elements. In addition, another criterion can be assessed, although it is a more liberal one [60]. For each construct, we also examined if loadings are greater than all of its cross-loadings [61,68]. This criterion is also met, as seen in Multimedia Appendix 2.

**Table 4:** Correlations^a^ and square root of average variance extracted^b^.

	PE^c^	EE^d^	SI^e^	FC^f^	HM^g^	PV^h^	HT^i^	SP^j^	BI^k^	UB^l^	Age	Gender	CD^m^
PE	.91												
EE	.47	.89											
SI	.31	.24	.98										
FC	.25	.57	.23	.79									
HM	.47	.44	.31	.32	.94								
PV	.42	.33	.34	.26	.42	.94							
HT	.43	.29	.55	.26	.48	.46	.81						
SP	.04	-.08	.15	-.06	.08	.08	.16	.72					
BI	.50	.43	.43	.29	.44	.35	.61	.17	.91				
UB	.23	.18	.39	.24	.17	.23	.41	.10	.44	N/A^n^			
Age	-.01	-.04	.13	-.03	-.01	.08	.09	.08	.08	.20	N/A		
Gender	-.02	-.04	.05	0	-.08	.05	0	.05	-.03	0	.11	N/A	
CD	-.08	-.10	.02	-.08	-.06	-.02	.03	.24	.01	.13	.18	.09	N/A

^a^Off-diagonal elements are correlations.

^b^Diagonal elements are square roots of average variance extracted.

^c^PE: performance expectancy.

^d^EE: effort expectancy.

^e^SI: social influence.

^f^FC: facilitating conditions.

^g^HM: hedonic motivation.

^h^PV: price value.

^i^HT: habit.

^j^SP: self-perception.

^k^BI: behavioral intention.

^l^UB: use behavior.

^m^CD: chronic disability.

^n^N/A: not applicable, because they are not reflective constructs.

Use, which was modelled using four formative indicators, is evaluated by specific quality criteria related to formative indicators. As seen in Table 5, the variance inflation factors are all below 5, suggesting that multi-collinearity is not an issue [64]. In addition, the indicators comply with the criterion of being statistically significant or, if not significant, its outer loading must be higher than .50 [64].

**Table 5:** Formative indicators’ quality criteria.

Indicators	VIF^a^	Weights	*t* (weights)	Outer loadings	*t* (loadings)
UB1^b^	2.609	.861	4.70^c^	.949	21.08^c^
UB2	1.707	.354	2.27^d^	.746	8.41^c^
UB3	3.237	.127	0.57	.741	8.46^c^
UB4	2.472	-.329	1.66	.543	4.50^c^

^a^VIF: variance inflation factor.

^b^UB: use behavior.

^c^ *P*<.01.

^d^ *P*<.05.

In sum, all assessments are satisfactory. This means that the constructs can be used to test the conceptual model.

### Structural Model

**Figure 5:**
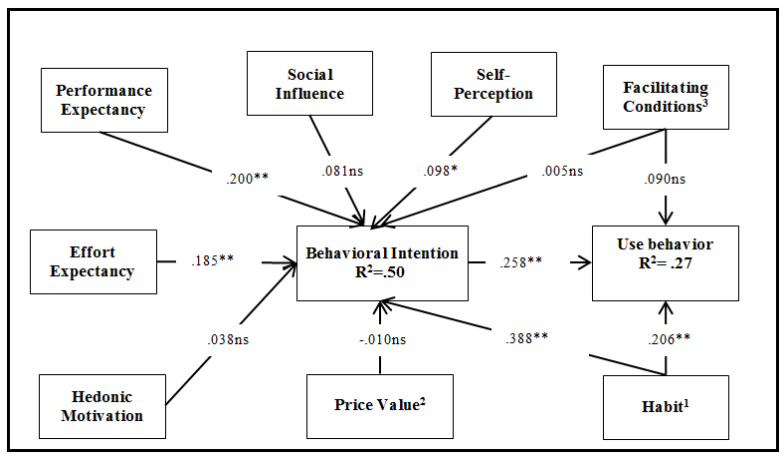
Structural model results. Notes: 1. Moderated by age or gender; 2. Moderated by age; 3. Moderated by chronic disability on use; *P<.01; **P<.05; ns: nonsignificant.

The structural model path significance levels were estimated using a bootstrap with 5000 iterations of resampling to obtain the highest possible consistency in the results. The *R* ^2^ was used to evaluate the structural model. Overall, the model explains 49.7% and 26.8% of the variance in behavioral intention and use behavior, respectively (see Figure 5).

Table 6 presents a summary of all the hypotheses tested and their support (or not) based on statistical tests. As Table 6 shows, the predictors of behavioral intention are performance expectancy (beta=.200; *t*=3.619), effort expectancy (beta =.185; *t*=2.907), habit (beta =.388; *t*=7.320), and self-perception (beta = .098; *t*=2.285). The predictors of technology use behavior are habit (beta =.206; *t*=2.752) and behavioral intention (beta =.258; *t*=4.036). Age also has a positive and significant effect on use behavior. This finding suggests that older individuals use EHR portal technologies more than do younger individuals.

We also tested the mediating role of behavioral intention between the independent variables and use behavior (see Table 7). To test if behavior intention mediated the independent variables on use behavior, we followed the Preacher and Hayes [64] approach. Initially, we check if only direct effects—without mediator (ie, behavior intention)—are statistically significant in explaining use behavior. Based on this (Step 1) we concluded that habit, facilitating conditions, and social influence are statistically significant, meaning that any of these factors might mediate behavior intention. Then in Step 2, we include the mediator variable (ie, behavior intention) in order to test if indirect effect of habit, facilitating conditions, or social influence are significant on use behavior. Only the indirect effect of habit is statistically significant (*P*<.01; *t*=3.472). Because of this fact, we compute the variance accounted for (VAF). The VAF is .38, meaning that behavior intention is a partial mediator of habit on use behavior [64]. Another important finding from this analysis is that in future studies it may be worth including a new relationship between social influence and use behavior, supported by a good literature background. This relationship is not foreseen in the UTAUT2 model.

**Table 6:** Summary of findings regarding hypotheses.

Dependent variables	Independent variables	Hypotheses (H)	Beta	*t*	*R* ^2^
Behavioral intention					49.7%
	PE^a^	H1 (supported)	.200	3.619^l^	
	EE^b^	H2 (supported)	.185	2.907^l^	
	SI^c^	H3 (not supported)	.081	1.544	
	FC^d^	H4 (a) (not supported)	.005	0.112	
	HM^e^	H5 (not supported)	.038	0.678	
	PV^f^	N/A^g^	-.010	0.203	
	PV x age	H6 (not supported)	.026	0.563	
	HT^h^	N/A	.388	7.320^l^	
	HT x age	H7 (a1) (not supported)	.033	0.584	
	HT x gender	H7 (a2) (not supported)	.010	0.183	
	SP^i^	H8 (supported)	.098	2.285^m^	
	Age	N/A	.065	1.408	
	Gender	N/A	.052	0.454	
	Gender x age	N/A	-.087	0.078	
	CD^j^	N/A	-.002	0.049	
Use behavior					26.8%
	FC		.090	1.755	
	FC x CD	H4 (b) (not supported)	.076	0.391	
	HT	N/A	.206	2.752^l^	
	HT x age	H7 (b1) (not supported)	.060	0.621	
	HT x gender	H7 (b2) (not supported)	.066	0.704	
	BI^k^	H9 (supported)	.258	4.036^l^	
	Age	N/A	.170	2.387^m^	
	Gender	N/A	-.013	0.092	
	Gender x age	N/A	.005	0.031	
	CD	N/A	-.081	0.476	

^a^PE: performance expectancy.

^b^EE: effort expectancy.

^c^SI: social influence.

^d^FC: facilitating conditions.

^e^HM: hedonic motivation.

^f^PV: price value.

^g^N/A: not applicable.

^h^HT: habit.

^i^SP: self-perception.

^j^CD: chronic disability.

^k^BI: behavioral intention.

^l^ *P*<.01.

^m^ *P*<.05.

**Table 7:** Mediating role of behavior intention on independent variables.

Step 1			Step 2			VAF^a^
Paths	Beta	*t*	Paths	Beta	*t*	
			PE^b^→BI^c^	.200	3.673^l^	
			EE^d^→BI	.188	2.844^l^	
			SI^e^→BI	.082	1.616	
			FC^f^→BI	.007	0.161	
			HM^g^→BI	.036	0.659	
			PV^h^→BI	-.007	0.131	
			HT^i^→BI	.392	7.313^l^	
			SP^j^→BI	.105	2.521^m^	
PE→UB^k^	.075	1.281	PE→UB	.067	1.125	
EE→UB	-.023	0.481	EE→UB	-.026	0.451	
SI→UB	.223	3.733^l^	SI→UB	.228	3.389^l^	
FC→UB	.124	2.609^l^	FC→UB	.132	2.578^m^	
HM→UB	-.107	1.617	HM→UB	-.112	1.629	
PV→UB	.012	0.192	PV→UB	.019	0.312	
HT→UB	.278	3.733^l^	HT→UB	.276	3.801^l^	
SP→UB	.065	1.122	SP→UB	.050	0.869	
			BI→UB	.271	3.746^l^	
			(FC→BI)×(BI→UB)	.003	0.256	
			(SI→ BI)×(BI→UB)	.021	1.390	
			(HT→BI)×(BI→UB)	.106	3.472^l^	.38

^a^VAF: variance accounted for.

^b^PE: performance expectancy.

^c^BI: behavioral intention.

^d^EE: effort expectancy.

^e^SI: social influence.

^f^FC: facilitating conditions.

^g^HM: hedonic motivation.

^h^PV: price value.

^i^HT: habit.

^j^SP: self-perception.

^k^UB: use behavior.

^l^ *P*<.01.

^m^ *P*<.05.

## Discussion

### Principal Findings

The results suggest that using our research model in a health-related area—EHR portal acceptance by health care consumers—yields good results, explaining 49.7% of the variance on behavioral intention and 26.8% of the variance in technology use [2]. The most important contributors with significant impact on behavioral intention are performance expectancy, effort expectancy, habit, and self-perception. The predictors of use behavior are habit and behavioral intention. The inclusion of a specific construct—self-perception—related to the health care consumer area had a significant impact on understanding the adoption of EHR portals, revealing the usefulness of integrating it into our research model. Age also had a positive and significant effect on technology use. This finding suggests that older individuals use EHR portal technologies more than do younger individuals, a belief that is found in the literature. There, it is mentioned that as age increases, the need for health care services also increases, and that this is reflected in more frequent access to health care services [8,69]. Our results were not able to support the finding that patients with chronic illness or disability are more likely to use EHR portals if they have the resources and support available. Our study had a lower proportion of people who mentioned having a chronic disability or illness compared with other studies [17,44]. This fact, together with the fact that our sample was also younger than those from other studies [17,44] and previous findings that older people usually require more support in using technologies [17,21,44], may explain why chronic disability did not achieve statistical significance as a moderator.

### Theoretical Implications

Concerning our results, some of our hypotheses were supported and others not; both H1 and H2 were supported. In studies that have addressed similar problems, including those studying patient portals [19,30,31], both performance and effort expectancy, originally from TAM [36], also had a significant positive impact. In our study, social influence did not show a significant effect on behavioral intention, thereby not supporting H3. Although the literature mentions the potential impact of social influence on the adoption of eHealth technologies [20,52], another recent study using UTAUT2 in health and fitness apps found no significant impact of social influence on behavioral intention [40], which is aligned with our study results. The rejection of the facilitating conditions hypothesis, H4 (a), suggests that the subjects in our sample consider that the resources or knowledge to use EHR portals are not an issue. This can be explained by the facility of having access to a computer and the Internet [4,12] and agrees with recent literature findings in eHealth [40].

Our results were also not able to confirm that patients with chronic illness or disability are more likely to use EHR portals if they have the resources and support available, as stated in H4 (b). This stands at odds with findings reported in the literature [17,44]. Earlier studies that addressed the concept behind H4 (b) included older people and those with a higher proportion of chronic disease or disability in the sample [17,44]. This may account for the difference in the results between our study and those reported in the literature. Future studies could address the degree or type of chronic disability.

Hedonic motivation also has no significant impact on behavioral intention (H5). Hedonic motivation is defined as intrinsic motivation (eg, enjoyment) for using EHR portals. Patients seem not to perceive the use of EHR portals as an enjoyment. This is probably because much of the use of portals is driven by the presence of a disease or a health problem, and the need for the portal is associated with that unfortunate fact—something that does not promote enjoyment [53,70]. Hedonic motivation had a positive impact on behavior intention in an eHealth study about health and fitness apps that promote balanced lifestyles [40]. These apps potentially have a greater impact on a person's hedonic motivation than the motives leading patients to use EHR portals. H6 was not verified. In Europe, access to the majority of eHealth services is free of charge [1,9], so the value that is given to the patients is to enable them to perform certain tasks more effectively online. Unfortunately, that fact is not being perceived by the patients.

The impact of habit in behavioral intention and use behavior was not moderated by age or gender; H7 (a1), H7 (a2), H7 (b1), and H7 (b2) were therefore not supported. However, the construct habit has a significant impact on both behavioral intention and use behavior, in line with findings from literature that mention habit as a predictor of behavioral intention and use behavior [18,40]. Self-perception, a construct related to health care, has a significant impact on behavioral intention, supporting H8. People who have a greater perception that they have health problems are more likely to use EHR portals. Our study’s findings are in line with other studies in this regard [31,47]. H9—behavioral intention will positively influence use behavior—was also supported. Literature suggests that using specific eHealth platforms is preceded by the intention to use them [18,22,30,31,35,45].

Overall, we were able to demonstrate that habit, a construct specific to consumer technology acceptance, and self-perception, which is related to the area of knowledge we are testing, are both very important in understanding the acceptance of EHR portals. Specific tailor-made models that incorporate specific changes related to the study’s topic may be an effective option for studying complex areas of knowledge, such as IT health care.

### Managerial Implications

**Figure 6:**
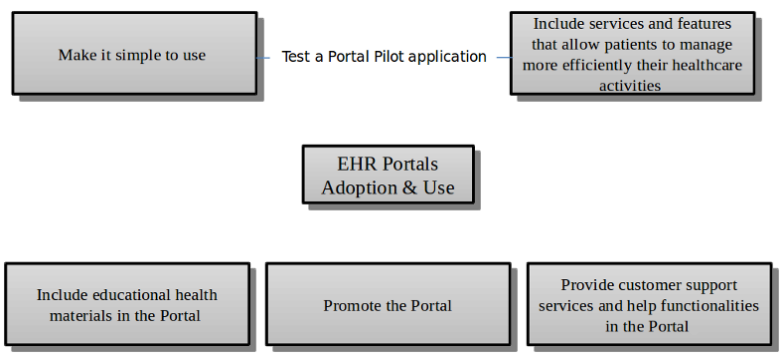
Managerial implications. EHR: electronic health record.

The findings of this study have valuable managerial implications for the conceptualization, design, and implementation of an EHR portal. We found that performance expectancy and effort expectancy have a significant impact on the adoption of EHR portals. Earlier studies using TAM identified these constructs as being relevant for the adoption of patient portals [30,41]. One of these studies adopted a qualitative TAM approach to evaluate patient portals [41], and the opinion of health care consumers in that study was that the design of these platforms should be simple and easy to use [41]. It is very important when designing or redeploying an EHR portal to make it easy and simple to use, and we therefore suggest that a pilot application of the platform be tested by the potential users so that improvements can be made during the development stage to increase the platform’s acceptance [71,72]. Our results suggest that there is a significant impact of health care consumers’ habit on EHR portal use. In addition to the direct and automatic effect of habit on technology use, habit also operates as a stored intention path to influence behavior [18]. This demands greater marketing communication effort to strengthen both the stored intention and its link to behavior [18]. Promotional strategies should therefore be implemented not only on the Internet, but also in the health care institutions that the patient usually goes to [56]. Because habit has been defined as the extent to which people tend to perform behaviors automatically because of learning [18], it is critical that EHR portals have client support services to help users with the platform. Another important finding is that the construct that is specific to health care—self-perception—also has a significant impact on the intention to use EHR portals. Self-perception relates to the fact that the perceived, rather than the real, severity of the health complaint is the propelling force behind the action [45]. Health care interventions that make the patient more aware of her/his health condition(s) may also promote the use of the EHR portal. Having a population that is better educated and more aware about health status could lead to a greater adoption of eHealth services, especially EHR portals. Overall, the managerial implications mentioned here are important not only for increasing the adoption of EHR portals, but also for increasing the frequency of usage of current users, who in most cases are not frequent users (see Figure 4). Figure 6 summarizes the managerial implications.

### Limitations and Future Research

We acknowledge that this research is limited by the geographic location, as it pertains to only one country and to only a sample of educational institutions. According to the literature, the technology that we are studying—EHR portals—is being used by less than 7% of the total number of health care consumers or patients [5,7,56]. The literature also mentions that users and early adopters of these types of platforms are younger than the population average and have significantly higher education [19,43,59]. Using a sampling strategy suitable to low-prevalence populations [57,58], we focused our sampling on educational institutions, where our target population is more concentrated [57]. It is also common to find studies that evaluate eHealth portals, addressing the users of a particular portal [16,19,30]. This is also a good strategy to target rare populations, but is also potentially biased as it reflects the opinions of only the users of a certain portal [19,57]. Another important fact that we acknowledge as a limitation in this study is that we were not able to collect the answers at more than one point in time. As a result, we could not use experience as a moderator in this study. Difficulties targeting the user population and the sensitivity of the topic related to EHRs [2] contributed to this limitation. The impact of chronic disability/illness as a positive moderator of facilitating conditions to explain technology use—pointed out as a possibility in the literature [17,44]—was not detected in our study. Nevertheless, only a small proportion of our sample (14.4%) mentioned having a chronic disability or illness and we did not collect information about its type or degree. Future studies might investigate this issue in greater depth.

Regarding the model tested, the inclusion of a health-related construct with significant positive impact demonstrates that it is relevant and that its inclusion is warranted. It also reveals the value of adding specific constructs related to the area in which the technology is used to existing frameworks. For future studies, it may also be advantageous to include other constructs (eg, confidentiality) that are not specific to health care but which, according to the literature, may be influential in eHealth adoption [2,19], or new relationships such as the one between social influence and use behavior. Some constructs from UTAUT2, notably hedonic motivation, do not seem to be relevant for EHR portal adoption and, in fact, self-perception seems to be a better motivational predictor. Future studies may therefore exclude this construct in order to avoid adding redundant complexity to the model. Another interesting future contribution is to evaluate mediated moderation in the research model.

### Conclusions

EHR portal adoption is a new and growing field of study that is an important topic in government-level discussions in the European Union and the United States. In our study, we used a new model in which we identified key additional constructs and relationships based on the literature review that are specific to IT health care adoption and integrated them into UTAUT2. The research model was tested and was found to explain 49.7% of the variance in behavioral intention and 26.8% of the variance in EHR portal technology use. Of all the constructs tested, performance expectancy, effort expectancy, self-perception, and habit had the most significant effects on behavioral intention. Habit and behavioral intention had a significant effect on technology use. Two specific constructs—habit (consumer related) and self-perception (health care)—were very significant in explaining the adoption of EHR portals, showing how important it is to use specific adoption models that include constructs specific to the area. The impact of chronic disability as a moderator of facilitating conditions to explain use behavior was not supported in our study. Not only is the adoption of EHR portals still low, but most current users of these platforms use them only infrequently. We used the results obtained in this study to provide managerial insights that may increase the adoption and usage of EHR portals.

## Appendix

Multimedia Appendix 1 Questionnaire items.

Multimedia Appendix 2 Partial least squares (PLS) loadings and cross-loadings.

## Abbreviations

AVE: average variance extracted
BI: behavioral intention
CD: chronic disability
CFIP: concern for information privacy
CMS: Centers for Medicare & Medicaid Services
CR: composite reliability coefficient
EE: effort expectancy
EHR: electronic health record
ELM: elaboration likelihood model
epSOS: European Patients Smart Open Services
FC: facilitating conditions
H1: hypothesis 1
H2: hypothesis 2
H3: hypothesis 3
H4 (a): hypothesis 4 (a)
H4 (b): hypothesis 4 (b)
H5: hypothesis 5
H6: hypothesis 6
H7 (a1): hypothesis 7 (a1)
H7 (a2): hypothesis 7 (a2)
H7 (b1): hypothesis 7 (b1)
H7 (b2): hypothesis 7 (b2)
H8: hypothesis 8
H9: hypothesis 9
HBM: health belief model
HIT: health information technology
HM: hedonic motivation
HT: habit
ICT: information and communication technology
IM: integrated model
IMS: Information Management School
IS: information systems
IT: information technology
MM: motivational model
N/A: not applicable
ns: nonsignificant
PE: performance expectancy
PEOU: perceived ease of use
PLS: partial least squares
PU: perceived usefulness
PV: price value
SI: social influence
SP: self-perception
TAM: technology acceptance model
UB: use behavior
UTAUT: unified theory of acceptance and use of technology
UTAUT2: unified theory of acceptance and use of technology in a consumer context
VAF: variance accounted for
VIF: variance inflation factor
