# Supplementary material for: Electronic Health Record Patient Portal Adoption by Health Care Consumers: An Acceptance Model and Survey
Source: J Med Internet Res. 2016 Mar 2;18(3):e49. doi: 10.2196/jmir.5069 (PMC4795321; doi:10.2196/jmir.5069)
Supplement: Multimedia Appendix 1 [file jmir_v18i3e49_app1.pdf]

Multimedia Appendix 1. Questionnaire's items

The scales' items were measured on a seven-point Likert scale, ranging from "strongly disagree" (1) to "strongly agree" (7). Use was measured on a different scale (explained in the table below).

Construct	Code	Items	Reference
Performance Expectancy	PE1	Using EHR Portals will support critical aspects of my healthcare.	[30]
	PE2	Using EHR Portals will enhance my effectiveness in managing my healthcare.	
	PE3	Overall, EHR Portals will be useful in managing my healthcare.	
Effort Expectancy	EE1	Learning how to use EHR Portals is easy for me.	[18]
	EE2	My interaction with EHR Portals is clear and understandable.	
	EE3	I find EHR Portals easy to use.	
	EE4	It is easy for me to become skilful at using EHR Portals.	
Social Influence	SI1	People who are important to me think that I should use EHR Portals.	[18]
	SI2	People who influence my behaviour think that I should use EHR Portals.	
	SI3	People whose opinions that I value prefer that I use EHR Portals.	
Facilitating Conditions	FC1	I have the resources necessary to use EHR Portals.	[18]
	FC2	I have the knowledge necessary to use EHR Portals.	
	FC3	EHR Portals is compatible with other technologies I use.	
	FC4	I can get help from others when I have difficulties using EHR Portals.	
Hedonic Motivation	HM1	Using EHR Portals is fun.	[18]
	HM2	Using EHR Portals is enjoyable.	
	HM3	Using EHR Portals is very entertaining.	
Price Value	PV1	EHR Portals is reasonably priced.	[18]
	PV2	EHR Portals is a good value for the money.	
	PV3	At the current price, EHR Portals provides a good value.	
Habit	HT1	The use of EHR Portals has become a habit for me.	[18]
	HT2	I am addicted to using EHR Portals.	
	HT3	I must use EHR Portals.	
Self-Perception	SP1	Do you think your health complaints are serious?	[45]
	SP2	Do you think your health complaints have to do with a serious disease?	
	SP4	Do you think that you could have treated your health complaints yourself?	
	SP6	Do you need more information about your health complaints?	
Behavioural Intention	BI1	I intend to use EHR Portals.	[18]
	BI2	I intend to use EHR Portals in the next months.	
	BI3	I plan to use EHR Portals frequently.	
Use Behaviour		What is your actual frequency of use of the following EHR Portal services? (i) Never; to (vii) every time I need it.	[18]
	UB1	Management of Personal Information and communication with health providers.	
	UB2	Medical appointments schedule.	
	UB3	Check your own Electronic Health Record.	
	UB4	Request for medical prescription renewals.	

Introduction presented to respondents before the questionnaire started:

Electronic health record portals are based on applying information technologies and systems on health environments. These portals allow, for instance, to make medical appointments online, to access medical history, medication records, specialists' summaries, and laboratory results. The access to these services is made through a web page, and allows you, as a patient, to manage your medical records. Please answer the questionnaire only if you have prior knowledge and contact with electronic health record portals. When we mention "EHR Portals" in this questionnaire, it refers to electronic health record portals.
