# Supplementary material for: Electronic Health Record Patient Portal Adoption by Health Care Consumers: An Acceptance Model and Survey
Source: J Med Internet Res. 2016 Mar 2;18(3):e49. doi: 10.2196/jmir.5069 (PMC4795321; doi:10.2196/jmir.5069)
Supplement: Multimedia Appendix 2 [file jmir_v18i3e49_app2.pdf]

Multimedia Appendix 2. PLS loadings and cross-loadings

Construct	Item	BI	EE	FC	HT	SP	HM	PE	PV	SI
Behavioural Intention	BI1	**.89**	.47	.32	.58	.11	.45	.54	.33	.36
	BI2	**.94**	.37	.24	.54	.16	.38	.43	.31	.40
	BI3	**.91**	.32	.22	.56	.21	.36	.41	.33	.43
Effort Expectancy	EE1	.36	**.87**	.51	.19	-.10	.32	.35	.26	.15
	EE3	.40	**.91**	.50	.28	-.07	.44	.47	.32	.25
	EE4	.34	**.86**	.49	.29	-.08	.44	.40	.33	.24
	EE5	.40	**.91**	.52	.27	-.03	.37	.43	.28	.20
Facilitating conditions	FC1	.20	.42	**.80**	.14	-.05	.16	.17	.14	.09
	FC2	.24	.49	**.88**	.21	-.03	.23	.20	.23	.20
	FC3	.28	.53	**.84**	.19	-.06	.27	.27	.17	.15
	FC4	.18	.32	**.64**	.28	-.05	.34	.13	.27	.29
Habit	H1	.52	.23	.24	**.88**	.15	.33	.30	.42	.59
	H2	.40	.13	.16	**.81**	.18	.39	.24	.37	.44
	H3	.56	.34	.22	**.75**	.08	.46	.50	.32	.31
Self-Perception	SP1	.10	-.11	-.11	.06	**.80**	.03	.00	.04	.06
	SP2	.15	-.09	-.12	.11	**.85**	.01	.01	.03	.15
	SP4	.11	-.05	-.01	.13	**.54**	.07	-.02	.11	.14
	SP6	.13	.01	.07	.15	**.65**	.14	.11	.05	.07
Hedonic Motivation	HM1	.41	.37	.27	.46	.12	**.96**	.43	.41	.31
	HM2	.41	.50	.37	.44	.01	**.90**	.49	.37	.28
	HM3	.41	.38	.25	.45	.11	**.96**	.41	.40	.30
Performance Expectancy	PE1	.37	.39	.18	.30	.04	.39	**.86**	.26	.18
	PE2	.51	.45	.25	.42	.05	.46	**.95**	.29	.29
	PE3	.48	.44	.23	.45	.02	.44	**.92**	.32	.35
Price Value	PV1	.30	.27	.22	.37	.09	.34	.22	**.91**	.28
	PV2	.34	.34	.27	.45	.07	.43	.34	**.96**	.32
	PV3	.35	.33	.24	.46	.07	.41	.33	**.95**	.34
Social Influence	SI1	.41	.23	.22	.54	.14	.27	.31	.32	**.97**
	SI2	.42	.23	.22	.54	.15	.31	.30	.33	**.98**
	SI3	.44	.24	.23	.55	.14	.34	.30	.34	**.98**
